# International Technologies on Prevention and Treatment of Neurological and Psychiatric Diseases: Bibliometric Analysis of Patents

**DOI:** 10.2196/25238

**Published:** 2022-02-22

**Authors:** Fuhao Zheng, Ling Wang, Zhaonan Zeng, Siying Wu

**Affiliations:** 1 Shengli Clinical Medical College of Fujian Medical University Fuzhou China; 2 Center for Experimental Research in Clinical Medicine Fujian Provincial Hospital Fuzhou China; 3 Public Health School of Fujian Medical University Fuzhou China

**Keywords:** neurological diseases, psychiatric diseases, patent, bibliometric analysis, prevention, treatment

## Abstract

**Background:**

Neurological and psychiatric disorders are serious and expensive global public health problems. Therefore, exploring effective intervention technologies plays an important role in improving patients’ clinical symptoms and social functions, as well as reducing medical burden.

**Objective:**

The aim of this study is to analyze and summarize the key new technologies and innovative development trends witnessed globally for neurological illness and psychiatric disorders by mining the relevant patent data.

**Methods:**

A bibliometric analysis was conducted on patent applications, priority countries, main patentees, hot technologies, and other patent information on neurological and psychiatric disorders, revealing the current situation along with the trend of technology development in this field.

**Results:**

In recent years, inventions and innovations related to neurological and psychiatric diseases have become very active, with China being the largest patent priority country. Of the top patent holders, Visicu (headquartered in the United States) is the leader. The distribution of patent holders in China remains relatively scattered, with no monopoly organization at present. Global technologies on neurological illness and psychiatric disorders are mainly concentrated around A61B (diagnosis, surgery, and identification).

**Conclusions:**

This paper analyzed and summarized the key new technologies and global innovative development trends of neurological and psychiatric diseases by mining the relevant patent data, and provides practical references and research perspectives for the prevention and treatment of the aforesaid diseases.

## Introduction

Neurological diseases mainly include diseases of the central nervous system, peripheral nervous system, skeletal muscle, among others, whereas mental diseases pertain to obstacles in mental development, emotion, volition, and behavior, etc. Although neurological illness and psychiatric disorders [1-3] cross each other, they are neither fully inclusive nor completely distinguishable. Studies based on neurological and psychiatric diseases can promote the generation and progress of related technologies, which can also further improve the prevention, diagnosis, and treatment of these diseases. Since the mid-20th century, many countries have performed studies on neurological and psychiatric diseases [1-3]. The United States, the European Union, Japan, Australia, and other developed countries have commenced long-term projects and increased financial support for scientific studies on neurological illness and psychiatric disorders [1-3] by proposing special and strategic plans to constantly expand research (both studies and teams) in this area. The United States plays a leading role in the investigation of neurological and psychiatric diseases. The National Institute of Medicine (NIH) and the National Science Foundation (NSF) are the main management and funding agencies in this field. The most prominent area of research in the field is *brain science*. The “Brain Research Through Advancing Innovative Neurotechnologies” (BRAIN) focuses on the construction of brain maps and new related medical treatments [4,5]. European Union’s “Human Brain Project” (HBP) puts emphasis on simulating brain functions via supercomputer technologies to realize artificial intelligence [6]. The Brain/MINDS (Brain Mapping by Integrated Neurotechnologies for Disease Studies) Program of Japan is focused on establishing animal models of brain development and diseases for studying the treatment of neurological and psychiatric diseases [7]. The Australian Brain Initiative lays stress on developing new treatment methods, new drugs, medical equipment, and wearable technologies by revealing the abnormal brain mechanisms of neuropsychiatric diseases [8]. As for China, financial support for studies on neurological and psychiatric diseases mainly comes from the National Natural Science Foundation (NSFC), the National Key Research and Development Plan, the National Key Basic Research and Development Plan of the Ministry of Science and Technology (973 Plan), and the National High-tech Research and Development Plan (863 Plan). Chinese studies were mainly performed on basic neurobiology techniques, neuropsychiatric diseases, brainlike artificial intelligence, transformative neuroscience, and supporting platform construction. Besides, China has strengthened the cultivation and support of neurological and psychiatric medication, neurobiomedical engineering, and artificial intelligence industries [9].

Many of the aforementioned technological achievements have successfully clarified the causes of several neurological and psychiatric diseases and reported the prevention and treatment measures. For example, the development of neural networks, internet, and big data technologies has enabled the prevention and treatment of related diseases at both population and individual levels. Analysis of the technological achievements concerning neurological and psychiatric diseases worldwide can reveal the progress and achieved cure level of each country in this field. Patent databases, an important analysis object, include more than 90% of the latest and most detailed technical information, and thus can accurately reflect the overall situation and development trend of technological innovation in global neurological and psychiatric diseases. Moreover, by performing patent bibliometric analysis, the internal information about technologies on neurological and psychiatric diseases such as distribution structure, quantitative relationship, and change rules can be clarified. Therefore, this study applied patent bibliometric analysis to explore the global research status and development trend of neurological and psychiatric diseases to provide valuable references for promoting the prevention and treatment of neurological and psychiatric diseases from an information science perspective.

## Methods

### Data Sources

Information about technologies on neurological and psychiatric diseases (IPC A61B5, G16 and H04, LOC 24) was retrieved and collected by International Patent Classification (IPC) and Locarno Classification (LOC). The date of data retrieval and download was September 1, 2019, and a total of 328 valid relevant patents were obtained.

### Data Collection and Analysis

The search keywords were psychosis, rehabilitation, psychology, rehabilitation training, rehabilitation resource survey, mental illness, mental trauma, psychological care, alzheimer’s, schizo, autism, anxiety, depressed, paranoid, manic, commit suicide, and so on. The retrieval type was “or.” First, all patent data were converted into plain text format and directly imported into a professional data analyzer (Thomson Data Analyzer [TDA]). The data were then cleaned, integrated, and analyzed. We adopted patentometric analysis and visualization to investigate the global technology development trends in the field of neurological and psychiatric disorders based on the following: patent application, distribution of patent priority countries, major patentees, hot technologies, patent citations, and so on.

## Results

### Global Patent Application and Authorization

**Figure 1:**
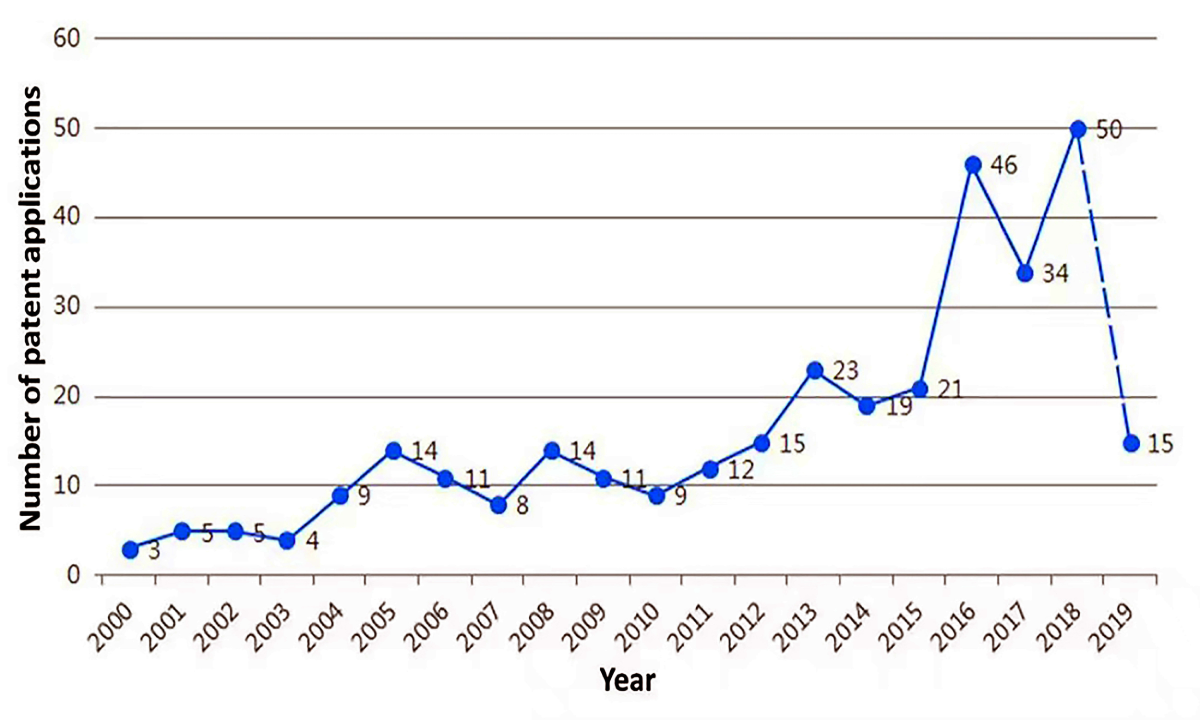
Trend chart of patent application.

A total of 328 patents related to neurological and psychiatric diseases were retrieved from 16 countries (regions), including 191 applications for inventions, 104 grants for inventions, 16 designs, and 17 utility models. Trend analysis was performed on the number of patent applications submitted annually (Figure 1). In the past 10 years, the number of patent applications related to the prevention and treatment of neurological and psychiatric disorders has generally increased year by year. However, the number of relevant patent applications was relatively small and the growth rate was relatively low (Figure 1). From 2015 onward, the growth rate began to accelerate and subsequently the number of applications submitted began to rise rapidly. From 2000 to 2004, the number of patent applications for neurological and psychiatric diseases was relatively low and was in the first stage (Figure 1). The second phase was from 2004 to 2015, and the number of applications increased to a certain extent. Since 2015, it has been in the third stage, and the number of applications has doubled from the previous stage.

### Analysis of Regional Competition Situation

**Figure 2:**
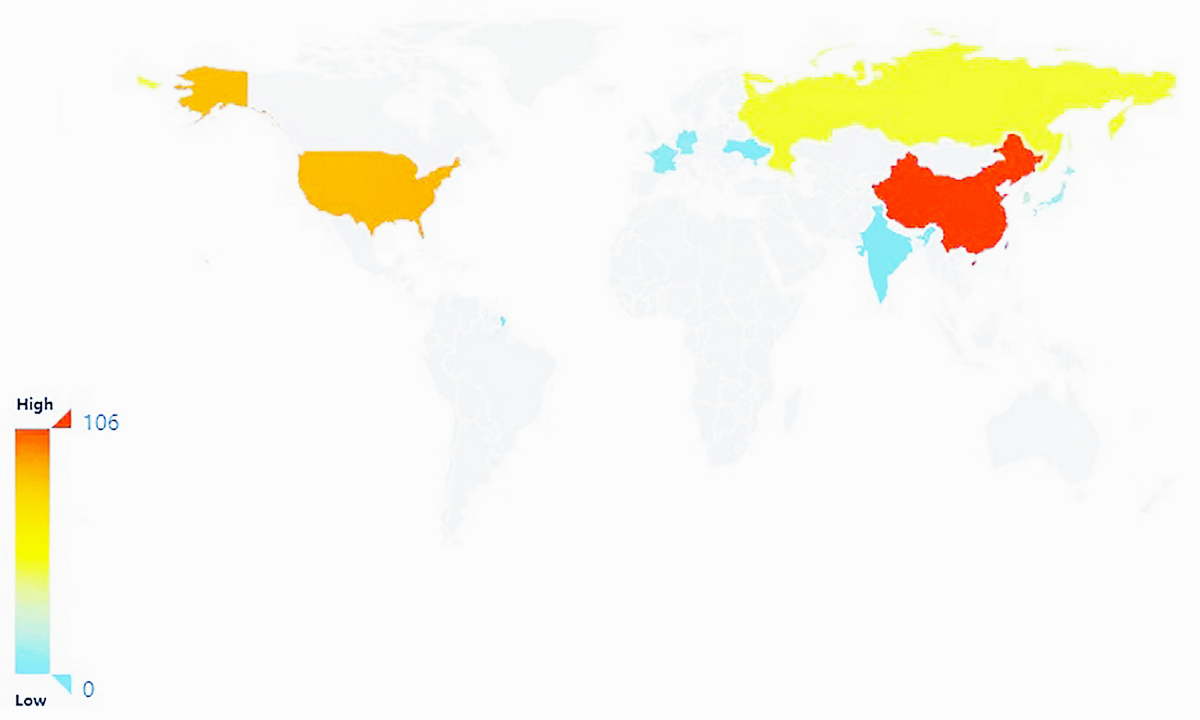
Global regional distribution of patent applications.

If an applicant files a patent application for his invention in one country for the first time and then files a patent application for the same subject matter in another country within the statutory period, the date of the first patent application shall be used as the filing date for the subsequent application according to the relevant law, so as to exclude the possibility of copying the patent in other countries and filing a pre-emptive application to obtain registration. By analyzing the distribution of patent priority countries, the attention and technical strength of various countries in this field can be deduced. Figure 2 shows the global regional distribution of the patent applications for neurological illness and psychiatric disorders. Since 2000, patent applications in this area have mainly come from China, the United States, and Russia. Among them, China and the United States are the front runners (or form the first echelon). The United States, which was the first country to introduce community-based prevention and treatment for patients with psychiatric disorders, developed community psychiatric rehabilitation technologies earlier than China and other countries. After more than 40 years of practice, community service has developed rapidly and achieved good results. China followed suite, occupying the second place, and currently has the largest number of patents. Russia, South Korea and Japan, as the second echelon, have submitted relatively few applications. France, Germany, India, Ukraine, the Soviet Union, Brazil, Canada, Europe, and the United Kingdom are in the third tier with even fewer applications. As for the distribution of patent priority countries, China leads the race and currently holds many core patents. As neurological and psychiatric disorders are common chronic noncommunicable diseases, and because China emphasizes on chronic disease management and continuous improvement of the medical security system, Chinese technologies for neurological and psychiatric diseases are showing a trend of continuous progress.

### Major Patentee

An analysis of those holding patents (ie, patent holders) on technologies useful for treating neurological and psychiatric disorders and the identification of major application agencies in this field can provide a strategic basis for interagency cooperation and competition. Visicu headquartered in Baltimore, Maryland, ranks second in the number of applications submitted and is the largest transferee of related patents in the world. As a clinical IT service provider and medical monitoring system developer, the company mainly develops and applies remote patient monitoring and diagnostic support technologies. Visicu was acquired by Royal Philips in 2008 (with the acquisition estimated to be 30 times the income before interest, taxes, depreciation, and amortization calculation). As can be seen from Table 1, Visicu’s applications are all US patents, most of which were filed in 2005 [10-19]. The major applications were A61B (diagnosis, surgery, and identification), G06F (electric digital data processing), and G08B (signaling or calling systems, order telegraphs, and alarm systems), which refer to electric digital data processing for diagnosis or surgery, and identification of medical and hygienic human necessities. Those are basically consistent with the technical distribution of relevant US patents.

**Table 1:** Patent form of Visicu (United States).

Patent name	Publication number	Application date	Reference
Remote command center for patient monitoring	US8175895B2	March 4, 2005	[10]
System for providing expert care to a basic care medical facility from a remote location	US7991625B2	May 31, 2006	[11]
System and method for displaying a health status of hospitalized patients	US7433827B2	February 18, 2005	[12]
Using predictive models to continuously update a treatment plan for a patient in a health care location	US7395216B2	May 31, 2006	[13]
System and method for displaying a health status of hospitalized patients	US20060161459A9	March 31, 2005	[14]
System and method for standardizing care in a hospital environment	US20060122869A9	February 18, 2005	[15]
Remote command center for patient monitoring relationship to other applications	US20060085229A9	March 4, 2005	[16]
System and method for displaying a health status of hospitalized patients	US20050187796A1	March 31, 2005	[17]
Remote command center for patient monitoring relationship to other applications	US20050177400A1	March 4, 2005	[18]
System and method for standardizing care in a hospital environment	US20050159987A1	February 18, 2005	[19]

The most frequently cited patent of Visicu is US7395216B2 [13], which uses predictive models to continuously update treatment plans for patients in different health care locations. The system comprises a database of patient data elements indicative of a medical condition associated with a patient. A predictive model is applied to patient assessment data and used to prepare a treatment plan. A rules engine applies a patient rule consistent with the treatment plan to selected data elements stored in the database to produce an output indicative of a change in the medical condition of the patient. The output from the rules engine is used to determine whether intervention is warranted. The predictive model is applied continuously to determine whether to update the treatment plan and, if necessary, the patient rule.

### Analysis of Technological Competition Situation

#### Analysis of Global Patent Hotspots

**Figure 3:**
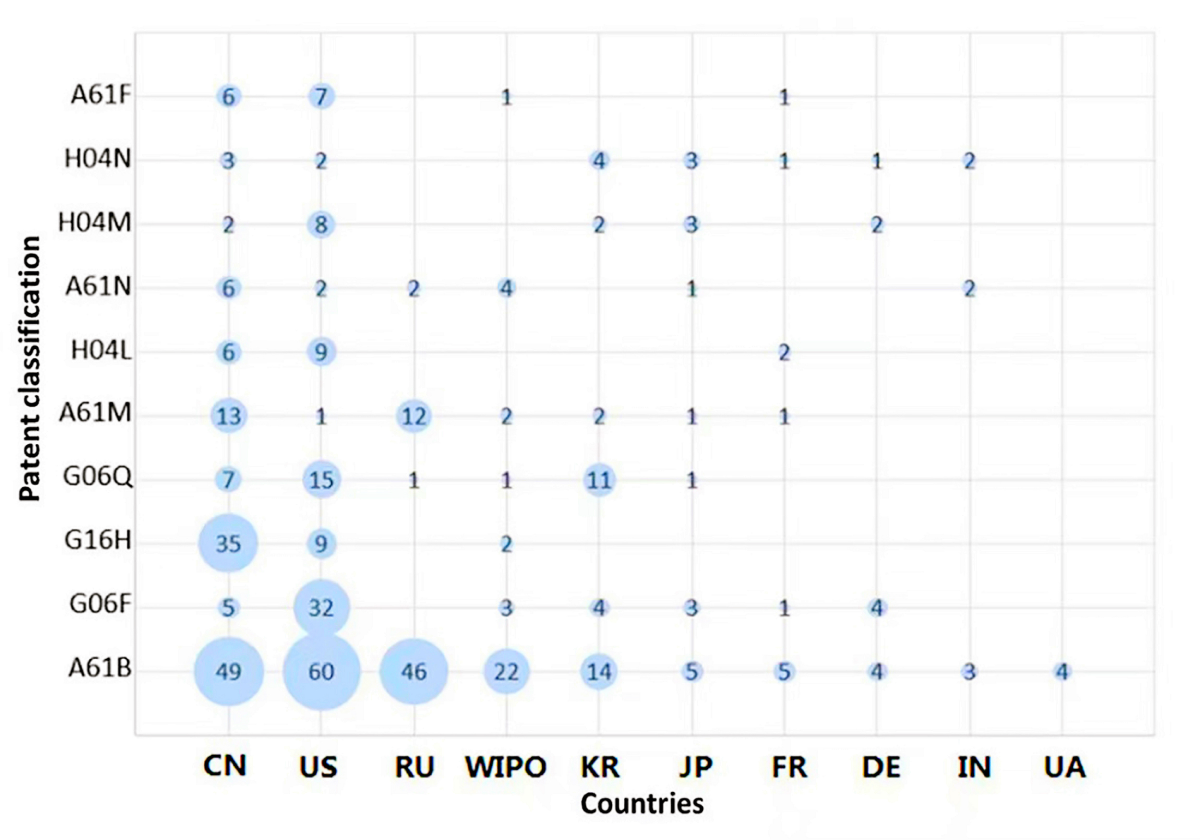
Patent technical distribution. CN, China; DE, Germany; FR, France; IN, India; JP, Japan; KR, Korea; RU, Russia; UA, Ukraine; US, United States; WIPO, World Intellectual Property Organization.

An analysis of the hotspots of patents related to neurological and psychiatric diseases indicated that researchers in the field are making continuous efforts to identifying new therapies and drugs, to further improve the effect of treatment and reduce the recurrence rate. Among the patents related to neurological illness and psychiatric disorders, most countries, especially the United States, have focused on A61B (ie, diagnosis, surgery, and identification; Figure 3). US A61B patents have been cited and transferred more frequently than those of other countries. For example, the patent named *Medical Emergency Alert System and Method* applied by Hwang et al [20] has been cited 286 times in various patents submitted by different countries. The medical emergency reporting system and its methodology utilizes a wearable monitoring device to continuously monitor key physiological parameters of a person, and when measurements exceed programmed threshold levels, it will automatically issue a medical emergency alert along with location information to a remote monitoring center via a wireless network and the internet for immediate local response. This system also provides manual emergency alert activation, continuous updates with key physiological measurements to the emergency response personnel along with the medical history of the individual as well as redundancy in emergency alert reporting and malfunction diagnosis to ensure accuracy, immediacy, and reliability for the person that requires medical assistance.

The US patents also concentrate on electric digital data processing (G06F), mainly the technologies related to internet big data. Among the US G06F patents, the latest trend pertains to computer-aided patient navigation and information systems. Such a system scans a patient’s electronic and oral communication to determine the medical needs of the patient, and then displays relevant information to appropriate medical personnel who can immediately advise the patient of the most appropriate source of medical assistance based on the identified symptoms.

China pays increased attention to the G16H (2018) class of patents, which pertains to psychotherapy research, and involves mainly psychotherapy, self-training, or computer-aided diagnosis. For example, the Medical Expert System [21], which comprises a mental retardation drug recommendation method and a system based on subtype classification of mental disorders, applied by Fudan University utilizes artificial intelligence and machine learning techniques to classify mental disorders by data mining and analysis of mental disorder diagnosis and treatment scale. The method specifically includes preliminary disease judgment, biclustered subtype classification, a posttreatment evaluation prediction model, and schizophrenia drug recommendations. The method can realize accurate and objective disease auxiliary diagnosis and treatment. Another example is Personal Health Risk Assessment [22], which includes a system and a device for evaluating the health status and rehabilitation progress. It is able to acquire physiological and psychological data, and then respectively perform feature selection/extraction on the data by mining and machine learning technologies. This method can provide more accurate health state evaluation, along with more rapid and effective treatment. Besides, there is information and communication technology. The Shenzhen Nanshan District Chronic Disease Prevention and Treatment Hospital has applied for a quality management system of community mental health service based on big data analysis. The system comprises an internet terminal module for mental health service, work management, and health education; a mental health service module for doctor follow-up, medical referral, and patient consultation; and a work management module for supervision and quality control. All kinds of information are collected into the subsystem and sent to the general system. Subsequently, the general system comprehensively evaluates the quality of community mental health services based on these big data. This tool assists doctors and assesses their work, to more comprehensively check for deficiencies and continuously improve mental health services.

#### Analysis of Global Application Types

The global patents for neurological and psychiatric disorders were mainly invention patents (Multimedia Appendix 1), which shows that the novelty and creativity of the patents in this field are relatively high and the quality of the patents is quite good.

Chinese invention patent grants related to neurological and psychiatric disorders were relatively few (Table 2). This is because the related technologies in China have only begun to sprout in recent years. Those concerned have just begun to apply for patent filing, and there is not enough time for examination and authorization. However, from the analysis of the number of applications and authorizations for invention patents in the United States, we can see that, after a period of development, the number of grants in the United States has started to increase. By contrast, Russia has a very large number of grants and a relatively high authorization rate.

**Table 2:** Types of global patents on neurological and psychiatric diseases.

Countries and regions	Invention/patent application, n	Invention patent grants, n	Design patents, n	Utility models, n
China	72	9	16	12
United States	52	33	0	0
Russia	4	40	0	2
World Intellectual Property Organization	24	0	0	0
Korea	10	12	0	0
Japan	8	3	0	0
France	4	3	0	0
Germany	5	1	0	0
India	4	1	0	0
Ukraine	0	2	0	2
Union of Soviet Socialist Republics	3	0	0	0
Brazil	1	0	0	1
Canada	2	0	0	0
European Patent Office	1	0	0	0
United Kingdom	1	0	0	0

Besides invention patents, China, Russia, Ukraine, Brazil, and other countries (regions) have utility model patents, but their number is very small, which indicates that most technologies in this field are protected by invention patents. Because this field is mainly concerned with methods such as medicine, the relevant patents are difficult to be protected with utility models. Moreover, China has design patents on equipment and instruments.

#### Hot Technology Analysis

**Figure 4:**
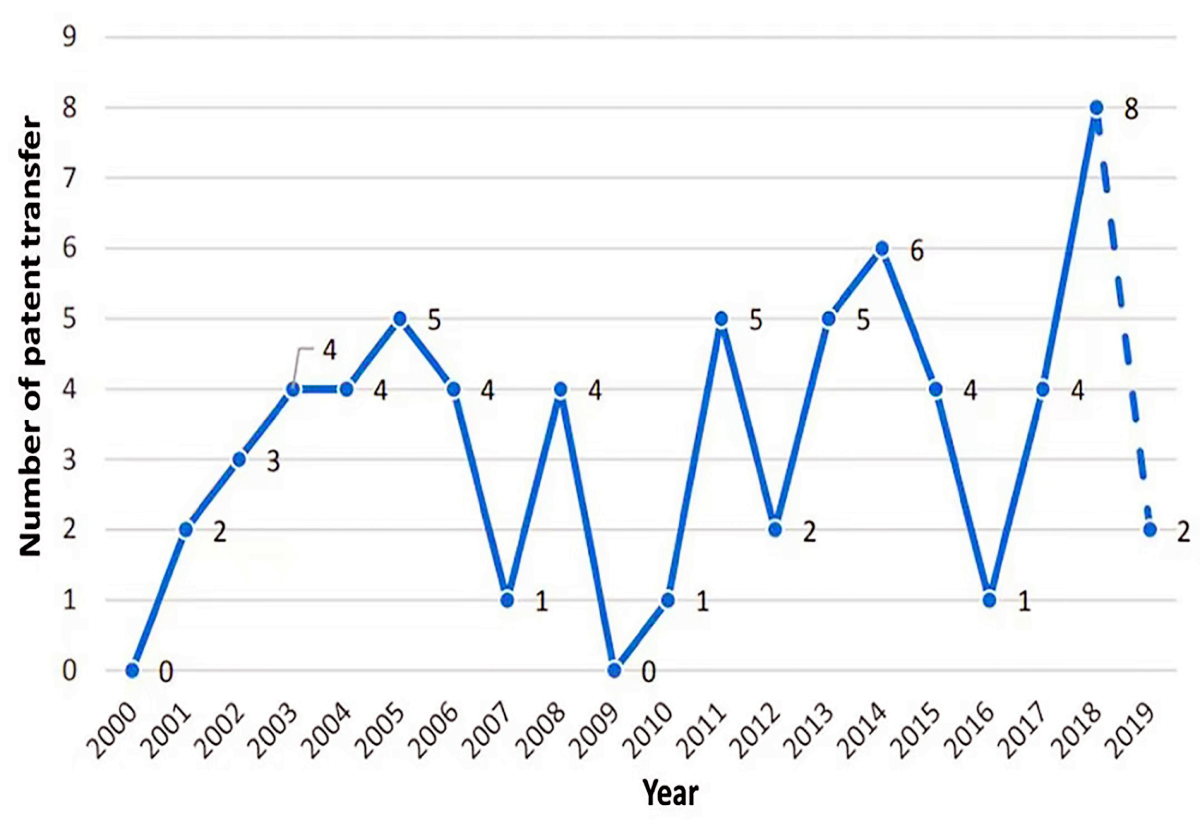
Global trend of patent transfer.

Generally, the higher the value of a patent, the more it will be transferred, and the more likely it will involve hot technologies in this field. The global trend of patent transfer is shown in Figure 4. Although patents related to neurological and psychiatric diseases have been transferred since the beginning of its application, the overall transfer trend remains stable. The number of transferred patents has fluctuated within 10, involving 65 transfers.

As can be seen from the global transferor ranking (Multimedia Appendix 2), Breslow and Rosenfeld, both of whom are medical doctors, have transferred the most relevant patents in the field of neurological and psychiatric disorders. Most of their transferred patents are methods for analyzing patient information and predicting treatment plans, including predictive models for treatment plan, methods for displaying patient health status, remote command centers for patient monitoring, among others.

Visicu has received 9 patents, which is the largest assignee (Multimedia Appendix 3), and also an important applicant in this field.

### Analysis of Technology Life Cycle

**Figure 5:**
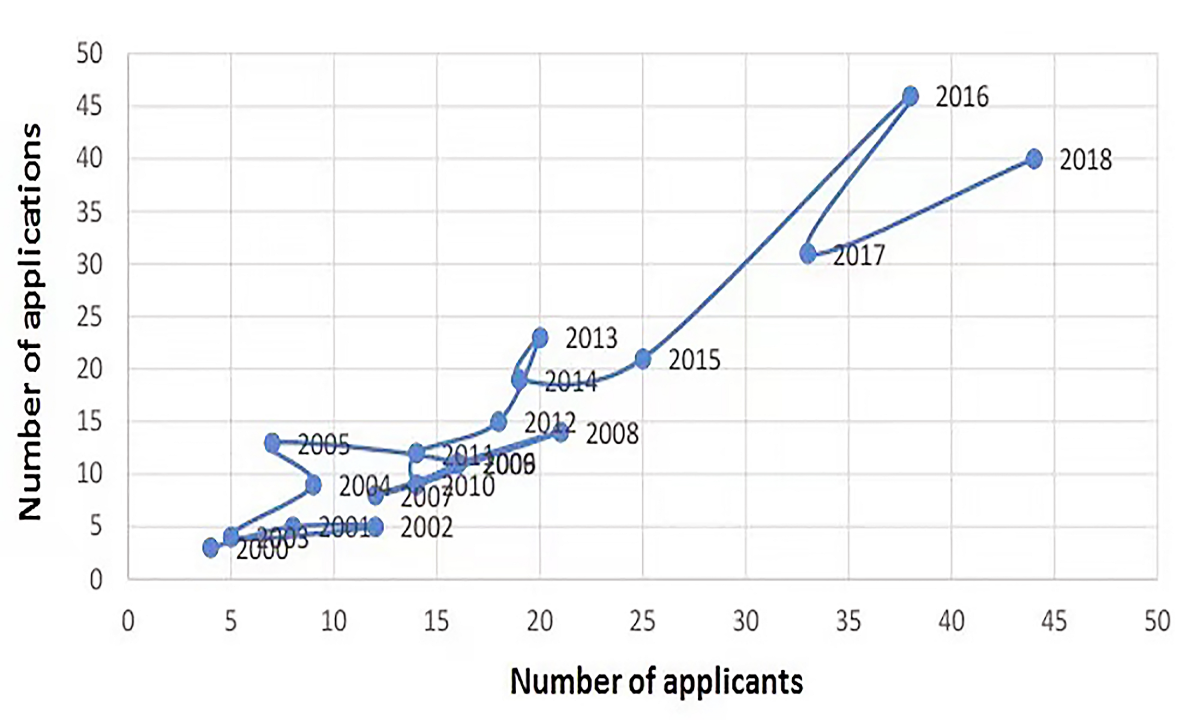
Life cycle diagram of patents.

According to the life cycle diagram of patents (Figure 5), since 2000, neurological and psychiatric disorders have not been an important area of focus. From 2000 to 2005, the number of patent applications and applicants in this field were relatively small, hovering up and down. At this time, few people showed solicitude for this field. Earlier patent applications in the United States related to methods and apparatuses for measuring indices of brain activity during motivational and emotional functions, which directly determines the composition and degree of the motivational and emotional brain circuitry response. This circuitry response answered questions focusing on normal and abnormal behavior, along with questions regarding the normal and abnormal functions of the circuitry. The results obtained by interrogating the motivational and emotional circuitry can be used for objectively measuring, in human or animal individuals, one’s preferences toward or responses to motivationally salient stimuli including but not limited to those that are internal or external, conscious or nonconscious, pharmacological or nonpharmacological therapies, disease- or non-disease–based processes, financial or nonfinancial, etc.

Between 2005 and 2015, the number of patent applicants has increased to a certain extent, but the number of patents has not risen significantly, suggesting that although more applicants paid attention to neurological and psychiatric diseases, the technology has not made much progress. Since 2015, with increasing public concerns for this field, and based on the development of big data and internet platform technologies, the patent field has begun to undergo certain transformations. Therefore, the number of applicants and applications for related patents has witnessed a sharp rise since. The most recent application (US20190200915) was on digital biomarkers for cognition and movement diseases or disorders, which assessed a cognition and movement disease or disorder in an individual suspected to have one [23]. A cognitive or fine motoric activity parameter was determined from a data set of activity measurements obtained from the individual using a mobile device. The determined activity parameter was compared with a reference, and the cognition and movement disease or disorder was assessed. A method for identifying whether an individual will benefit from a therapy for a cognition and movement disease or disorder was also disclosed. The steps just described are performed along with the step for identifying the individual as one who will benefit from the therapy if the cognition and movement disease or disorder is assessed. A mobile device was revealed as well, comprising a processor, at least one sensor, a database, and software that is tangibly embedded in the said device and when installed on the said device performs the disclosed methods.

Research and development on patents for neurological and psychiatric disorders are significantly influenced by government policies and the market. Therefore, the number of applications and applicants will grow each time when this field is favorably affected. This means that neurological and psychiatric diseases remain in the focus of scientific and technological development, and professionals are still very much interested in it.

## Discussion

### Principal Findings

China, as the country with the largest number of patents related to technologies on neurological and psychiatric diseases, has 107 related patents, which mainly focus on A61B and G16H. Jilin Longjin Technology Co., Ltd., the applicant with the most patents on this topic in the world, is headquartered in China. China has not only applied for many invention patents, but also applied for 11 utility model patents and 15 appearance designs. Most global utility models and appearance design patents for community psychiatric rehabilitation are in fact Chinese patents. However, the authorization rate of invention patents in China remains very low, which may be because most patents in China were only proposed after 2015 and are thus still in the substantive examination stage. Although China had put forward relevant theories later than the United States and other Western countries, it has caught up and currently occupies a large market. The number of patents for neurological illness and psychiatric disorders in the United States is second only to that in China, with a total of 85, but all of these are invention applications with a high authorization rate. Besides A61B, the United States focuses on G06F, which is slightly different from that of China. Russia closely follows the United States, being the third country with 46 related patents, which is only about half of the number of US patents. The per-capita patent applications of Russian applicants are extremely low, with only 1 applicant applying for 3 patents and the rest applying for only 1 patent. Furthermore, unlike China and the United States, Russia’s patents are mainly focused on medical diagnosis and medical devices. There are no patents involving mobile devices.

The global development of technologies on neurological and psychiatric disorders shows the following characteristics: (1) The technology is increasingly mature. The number of patent applications in recent years has remained at a high level. Inventions and innovations are very active, with China currently being the largest patent priority country. (2) European and American companies, with a strong independent innovation capability, have considerable advantages in the area of research and development. The distribution of patent holders in China remains relatively scattered, with no technology monopoly organization at present. It is thus necessary to strengthen the collaboration between production, teaching, and research. (3) The types of patent applications worldwide are mainly invention patents. A few countries such as China and Russia have applied for a small number of utility model patents. (4) Patents mainly pertain to A61B (ie, medical and hygienic fields). By 2011, the classification number G16H (ie, health care informatics) was added, and eventually patents about internet-related mobile terminal platforms began to appear. The number of applications has increased year by year. As a newly emerging technology, there is still however great room for its development. From the special perspective of patents, this paper investigated the key technologies and development directions of industries as well as the technology combination and technology investment trends of major competitors for enterprises or countries, so as to establish technology research and application strategies on neurological and psychiatric diseases.

This paper had certain limitations. Because of the large number of patents involving neurological and psychiatric diseases, it is impossible to make a detailed interpretation of the contents of each document. Therefore, this study focused on the key technical points, categories, and regions of the patents, and illustrated the layout of the patents based on the primary and secondary technical points of the cluster diagram.

### Conclusions

Based on this analysis, we concluded the following: (1) The United States, as a relatively mature country in the development of existing technologies, can focus its attention on further development in this area. (2) Technologies on neurological and psychiatric disorders in China have a low authorization rate for invention patents. It is thus suggested to improve the quality of Chinese patent applications and submit high-value patents. (3) In Russia, the number of patent applications on neurological and psychiatric diseases is generally small, but the authorization rate is extremely high, and therefore these need to be tracked.

## Appendix

Multimedia Appendix 1 Global patent type.

Multimedia Appendix 2 Global ranking of patent transferors.

Multimedia Appendix 3 Global ranking of patent assignees.

## Abbreviations

BRAIN: Brain Research Through Advancing Innovative Neurotechnologies
Brain/MINDS: Brain Mapping by Integrated Neurotechnologies for Disease Studies
HBP: Human Brain Project
IPC: International Patent Classification
LOC: Locarno Classification
NIH: National Institute of Medicine
NSF: National Science Foundation
NSFC: National Natural Science Foundation
TDA: Thomson Data Analyzer
