# Supplementary figures and images for: International Technologies on Prevention and Treatment of Neurological and Psychiatric Diseases: Bibliometric Analysis of Patents
Source: JMIR Ment Health. 2022 Feb 22;9(2):e25238. doi: 10.2196/25238 (PMC8905476; doi:10.2196/25238)

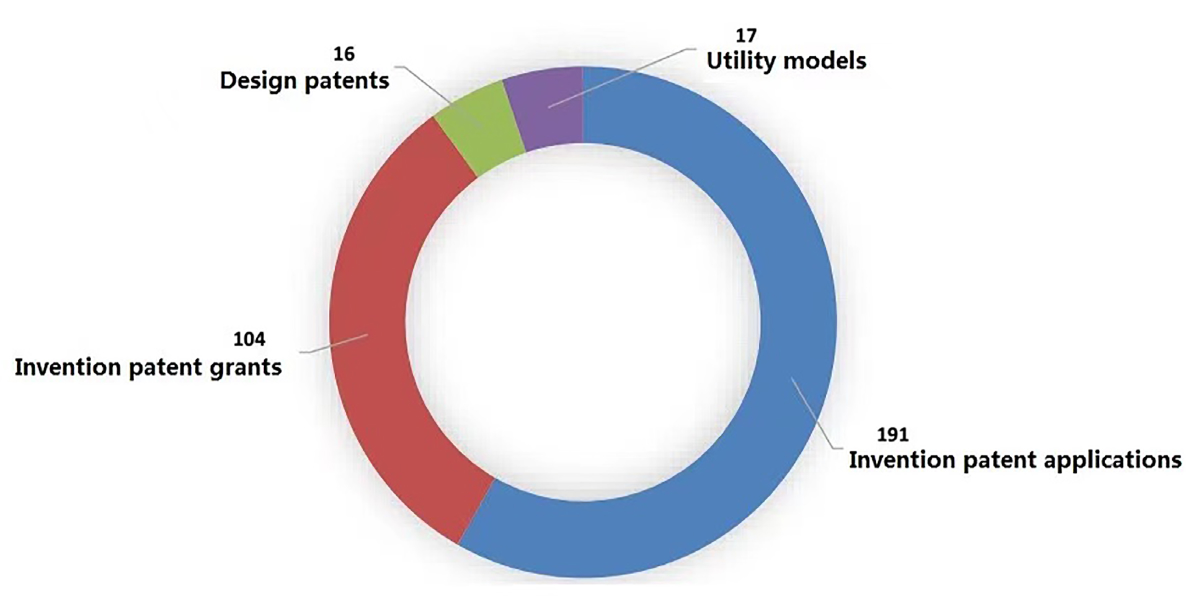

Supplement: Multimedia Appendix 1 [file mental_v9i2e25238_app1.png]

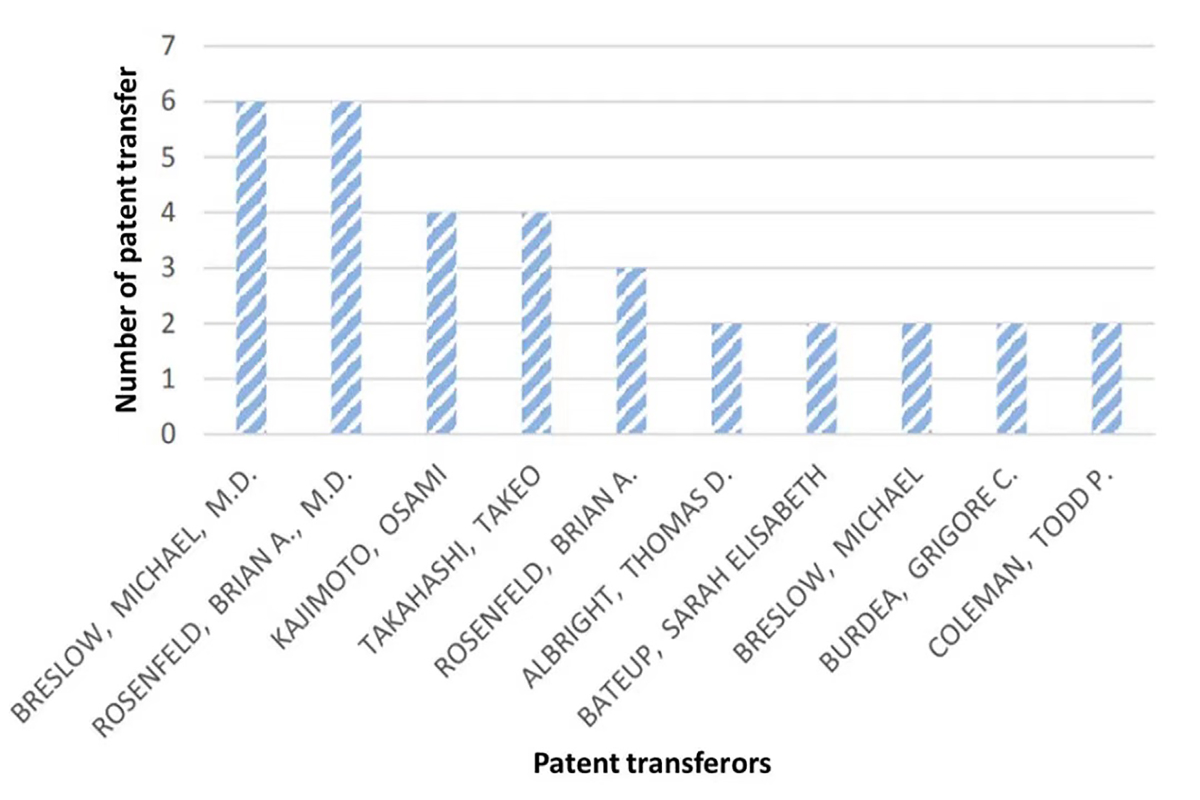

Supplement: Multimedia Appendix 2 [file mental_v9i2e25238_app2.png]

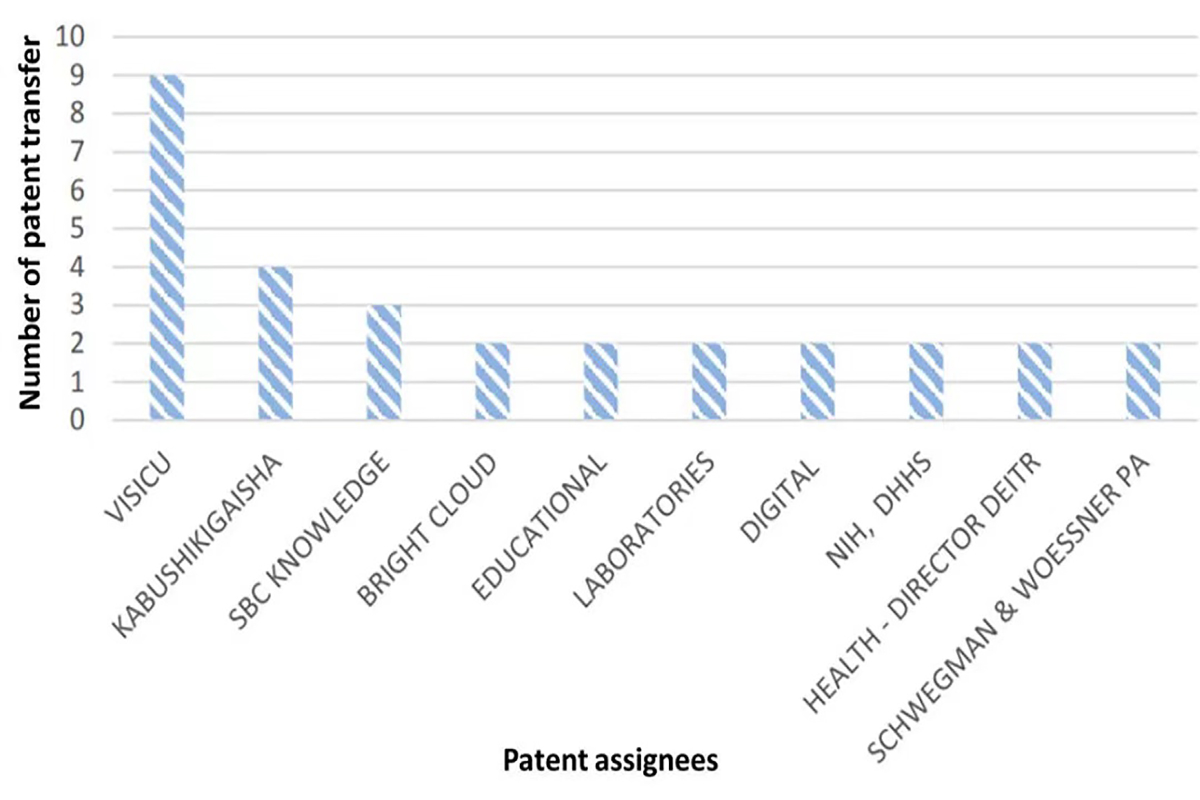

Supplement: Multimedia Appendix 3 [file mental_v9i2e25238_app3.png]
